# Relationships between Social Capital, Social Capital Satisfaction, Self-Esteem, and Depression among Elderly Urban Residents: Analysis of Secondary Survey Data

**DOI:** 10.3390/ijerph16081445

**Published:** 2019-04-23

**Authors:** Hyun Jin Lee, Dong Kun Lee, Wonkyong Song

**Affiliations:** 1 Interdisciplinary Program in Landscape Architecture, Seoul National University, Seoul 08826, Korea; hjlee411@snu.ac.kr; 2 Department of Landscape Architecture and Rural System Engineering, Research Institute of Agriculture and Life Sciences, Seoul National University, Seoul 08826, Korea; 3 Department of Landscape Architecture, Dankook University, 119, Dandae-ro, Dongnam-gu, Cheonan, Chungnam 330-714, Korea; wksong@dankook.ac.kr

**Keywords:** depression, elderly urban residents, mental health, self-esteem, social capital

## Abstract

The role that psychological variables play in depression among elderly urban residents has received little research attention. Therefore, the purpose of this study was to examine the relationships between social capital, social capital satisfaction, self-esteem, and depression among elderly urban residents. We used the responses provided by 701 elderly persons to scales assessing social capital (i.e., network, trust), social capital satisfaction, self-esteem, and depression, as part of the Korea Welfare Panel Study (KOWEPS). We conducted a confirmatory factor analysis and tested the validity of a proposed statistical model using structural equation modeling (SEM). The results showed that trust in social capital, social capital satisfaction, and self-esteem were significantly related to depression. Further, social capital satisfaction and self-esteem fully and partially mediated the relationship between trust and depression, respectively. These findings serve as an empirical base upon which social welfare policies can be founded that benefit elderly urban residents with weak social capital, low social capital satisfaction, and poor self-esteem.

## 1. Introduction

The urban environment promotes social disruption and renders its residents more vulnerable to mental health problems, such as anxiety and depression [1]. In 2017, 91.8% of the Korean population lived in cities due to rapid urbanization [2]. Further, the elderly urban population also rapidly increased from 56.4% in 1994 to 76.6% in 2014 [3]. The elderly are particularly susceptible to depression owing to both endogenous factors, such as physiological changes (e.g., reduced levels of Serotonin) [4] as well as exogenous factors such as reduced social networks resulting from downward mobility in socioeconomic status [5].

Depression in the elderly can adversely affect their likelihood of developing dementia [6,7] and committing suicide [8], thereby leading to increased social and economic costs [9]. The WHO reported that mental health is related to physical and social health and consequently burdens the health care system and impacts overall survival [10]. Therefore, the problem of depression among elderly urban residents is recognized as a social problem rather than as an individual-level problem; it is necessary to strengthen strategies that are aimed at preventing depression among the elderly. In recent years, pharmacological [11] and non-pharmacological [12] strategies have been made available worldwide to attenuate depression-related disorders and psychological impairment. Given the advantages of pharmacological evidence, both recent standard serotonin-reuptake inhibitors (SSRIs) and serotonin-norepinephrine reuptake inhibitors (SNRIs) [11] to date have demonstrated their efficacy and potential to prevent negative outcomes associated with this invalidating illness. Therefore, the correct and rapid recognition or treatment of this disabling condition is an absolute imperative for the global community. However, depression is frequently associated with cognitive dysfunction. When it comes to non-pharmacological options, the existence of a trend toward improvements in the neurocognitive profile with repetitive transcranial magnetic stimulation (rTMS) has been demonstrated [12]. Therefore, new available treatment strategies may be considered promising for cognitive enhancement in major depression in order to avoid or attenuate the likelihood of developing disabling conditions such as dementia.

On the other hand, several factors have been found to play a role in depression among the elderly, including physiological factors [4,13,14], health status [15,16,17], socioeconomic characteristics [8,15,18,19], and health behaviors (e.g., leisure activities, physical activity) [20,21,22,23]. These factors can be addressed by adequate economic welfare support policies. However, many reports suggest that various aspects of the social environment, such as social capital and psychological factors, are associated with depression in the elderly [24]. In a meta-analytic study, Kim and Son (2005) showed that depression in the elderly evidenced the highest mean correlation coefficient with psychological variables and the lowest mean correlation coefficient with demographic variables [24,25]. Specifically, correlation coefficients that emerged between depression and psychological variables (i.e., self-esteem, life satisfaction) yielded an average that was above 0.30. In other words, depression in the elderly showed a higher correlation with psychological variables than with demographic variables; it evidenced the strongest correlations with life satisfaction and self-esteem [25].

The findings of previous studies suggest that elderly people with high social capital, life satisfaction, and self-esteem tend to obtain low scores on measuring depression, making necessary policy efforts that are aimed at extending the social capital of the elderly. Social capital is defined as an individual’s social network that is built on mutual trust, agreed-upon standards, sharing, etc. [26,27,28,29]. It acts as a protective factor and plays an important role in improving mental health status and reducing the prevalence of mental health disorders [30,31]. Accordingly, previous studies have found that social capital and support significantly improve mental health [16,23,24,32,33,34,35]. Individual’s cognitive social capital is known to be a psychological factor of depression [30]; social support perceived by such social capital alleviates the loneliness and stress of the elderly [35]. It has also been reported that social capital facilitates subjective mental well-being and reduces psychological problems such as depressive symptomatology or anxiety [36,37]. At the same time, social capital is one predictor of individual life satisfaction [38]. Life satisfaction reflects the consequences of social conditions, norms, and interactions among social members. Therefore, positive perceptions of social capital and networks can be expressed as subjective satisfaction with the social environment and is a part of measurements of life satisfaction [39]. Life satisfaction is defined as the subjective and overall assessment of an individual’s life [40]. The variables that affect satisfaction with social environment include not only demographic characteristics but also social contact and relationships with others, such as friends or neighbors [41]. Life satisfaction is also expressed as quality of life or well-being and includes personal perceptions of socio-emotional functioning, social capital, well-being, and health status. Therefore, satisfaction with an individual’s cognitive social capital is included in life satisfaction [26].

Little research examined the psychological aspects of social capital that affect depression. Therefore, it is necessary to understand the mediating effects of psychological factors in the relationship between social capital and depression in order to help elderly urban residents who are vulnerable to mental health problems build effective social capital.

The primary aim of this study was to identify the differential sociodemographic characteristics of elderly urban residents who are vulnerable to low social capital satisfaction, poor self-esteem, and depression. A second objective was to examine the relationships between depression and psychological factors such as social capital, satisfaction with social capital, and self-esteem, among elderly urban residents. Finally, we aimed to use the findings to provide evidence-based recommendations that are aimed at alleviating depression among elderly urban residents.

In this study, the hypotheses for the research model were set as follows: (1) Social capital, social capital satisfaction, and self-esteem are directly related to depression; (2) social capital is directly related to social capital satisfaction and self-esteem; (3) social capital satisfaction and self-esteem can mediate the relationship between social capital and depression; and (4) self-esteem can mediate the relationship between social capital satisfaction and depression.

## 2. Materials and Methods

### 2.1. Data Collection and Research Participants

This cross-sectional study used data that were collected as part of the 12th edition of the Korea Welfare Panel Study (KOWEPS) in 2017 by the Ministry of Health and Welfare and the Korea Institute for Health and Social Affairs (KIHASA) and Seoul National University (SNU). The data contains information about the living conditions and welfare needs of each population group differing in age and income level for each year since 2006 [42]. This survey did not use personally identifiable information, and all participants signed an informed consent form before the survey [42,43]. In a survey, trained researchers directly visited each household that was selected from a nationwide stratified double sampling in order to collect data by means of interviews [42]. The raw data can be downloaded from the Welfare Panel’s website [43].

A total of 701 individuals (295 (42.1%) men and 406 (57.9%) women) who lived in urban environments and responded to all the survey questions were included in the present study. This sample consisted of 206 individuals (29.4%) between the ages of 66 and 70 years, 198 individuals (28.2%) between the ages of 71 and 75 years, 198 individuals (28.2%) between the ages of 76 and 80, and 99 individuals (14.1%) aged 81 years or older. With regard to educational level, 494 (70.4%) respondents endorsed the option, “Below primary school”. For household type, 453 individuals (64.6%) reported living in “elderly single-generational households”, 245 individuals (35.0%) reported living in “single-member households”, and 3 individuals (0.4%) reported living in “multi-generational households”. More than half of the participants perceived their subjective health to be poor (412 people (58.8%)). Further, 509 respondents (72.6%) were classified as “low income status”, based on the below 60% of median income. This study was approved for exempt status by the Institutional Review Board of our university (IRB No. E1903/003-009).

### 2.2. Instruments

#### 2.2.1. Depression

The brief 11-item Korean version of the Center for Epidemiologic Studies Depression Scale (11 CES-D) [44,45], translated by Jeon and Rhee [46], was used to measure depression in the KOWEPS [42]. After eliminating items that reduce reliability on account of low correlation coefficient between observed variables (Table A1) [47], the selected variables were entered into our research model (Table 1). The total depression score was obtained by the sum of all the items with reverse coding of relevant items. Responses were recorded on a four-point scale ranging from “extremely rare”, which is score of 1, to “most of the time”, which is score of 4. Scores on the test range from 7 through 28; high composite scores indicated a high degree of depressive symptomatology [42]. Reliability of the scale was estimated using Cronbach’s alpha, which was found to be 0.88 in this study.

#### 2.2.2. Social Capital

Questions assessing social capital related to family, friends and neighbor as sub scales in the KOWEPS [42]. However, in the present study, only the relationships with friends and neighbors were assessed using six items given the research purpose of considering the social environment. In this study, six items pertaining to the social capital of the elderly were divided in terms of their social networks (i.e., network capital) and their subjective perceptions of trust in their relationship with others (i.e., cognitive trust).

Network capital was assessed using the following three items: “There are special people (friends or neighbors) who can help in an emergency”. “There are special people (friends or neighbors) who make me comfortable”. and “There are friends or neighbors around to share joy and sorrow”. Responses can be recorded on a five-point Likert scale ranging from “strongly disagree”, which is score of 1, to “strongly agree”, which is score of 5; high composite scores indicate a strong sense of cognitive network capital. Cronbach’s alpha for this subscale was found to be 0.92 in the present study.

The measurement of cognitive trust was also confined to friends and neighbors. Cognitive trust was assessed using the following three items: “My friends or people around me try to help me.” “I can depend on friends or people around when I’m in trouble.” and “I can talk to friends or people around me about my problem”. Responses to each item can be recorded on a five-point Likert scale ranging from “strongly disagree”, which is score of 1, to “strongly agree”, which is score of 5; high composite scores indicate a strong sense of cognitive trust. The internal consistency of the three items, estimated using Cronbach’s alpha, was found to be 0.92 in the present study.

#### 2.2.3. Social Capital Satisfaction

In the KOWEPS data, the satisfaction scale assesses satisfaction with life domains such as health, family income, residential environment, occupation, family relationship, social relationship, and leisure [42]. In this study, three domains related to the social environment (social relationship, leisure, and overall) [48] were selected for our research model (Table 1). Each item can be rated on a five-point Likert scale ranging from “strongly disagree”, which is score of 1, to “strongly agree”, which is score of 5; high composite scores indicate high social capital satisfaction. Cronbach’s alpha for this scale was found to be 0.83 in the present study.

#### 2.2.4. Self-Esteem

The Korean version of the Rosenberg Self-esteem Scale (RSES) [49] was used to measure self-esteem in the KOWEPS [42]. After eliminating items that reduce reliability on account of low correlation coefficient between observed variables (Table A2) [47], the selected items were entered into our research model (Table 1). The scale scores are the sum of all the items. Items are rated on a four-point scale ranging from “strongly disagree”, which is score of 1, to “strongly agree”, which is score of 4; high composite scores indicate high self-esteem. The internal consistency of this scale, estimated using Cronbach’s alpha, was found to be 0.77 in the present study.

### 2.3. Statistical Analysis

Descriptive statistics were used to examine the sociodemographic characteristics of the participants. The chi-square test was used to investigate sociodemographic differences in social capital satisfaction, self-esteem, and depression. Analyses were conducted using version 18 of the PASW Statistics (SPSS Inc, Chicago, IL, USA).

Structural equation modeling (SEM) was used to examine the relationship between social capital and depression, social capital satisfaction, and self-esteem among elderly urban residents. Two steps were undertaken to test the validity of our model. First, we conducted a confirmatory factor analysis to assess whether the observed variables are valid indicators of the latent variables. Second, the goodness-of-fit of the model and the mediating effects of variables were tested using structural models [50]. To explore the association effects of psychological variables (i.e., social capital, social capital satisfaction, self-esteem) and depression, we conducted an analysis of the mediating effect of the SEM using the bootstrap verifying method [51].

The SEM model included both exogenous variables (i.e., social capital, measured in terms of network and trust) and endogenous variables (i.e., social capital satisfaction, self-esteem, and depression). Version 22 of AMOS (IBM, New York, NY, USA) for Windows was used to execute SEM and calculate the maximum likelihood estimates of the model parameters and the model fit indices. Goodness-of-fit of the measurement and structural models was examined using the following absolute fit indices [52,53]: Minimum value of the discrepancy function (CMIN: χ^2^), CMIN/df (Normed χ^2^) less than 2 or 3, Goodness-of-fit index (GFI) greater than 0.90, Root Mean Square Residual (RMR) less than 0.05, and Root Means Square Error of Approximation (RMSEA) less than 0.08. Additionally, the following relative fit indices [52,53]: Normed Fit Index (NFI), Tucker Lewis Index (TLI), and Comparative Fit Index (CFI) greater than 0.90 were also used.

## 3. Results

### 3.1. Sociodemographic Differences in Social Capital Satisfaction, Self-Esteem, and Depression

Table 2 shows sociodemographic differences in the dependent variables. Specifically, social capital satisfaction, self-esteem, and depression were significantly different across groups varying in household type, health status, and income status. However, no difference was found in self-esteem scores across groups differing in household type. Those individuals belonging to multi-generation households reported lower social capital satisfaction and self-esteem and greater depressive symptomatology than those individuals belonging to other household types. Participants with poor health also reported lower social capital satisfaction and self-esteem and greater depressive symptomatology than their healthier counterparts. Similar results were observed when groups differing in income status were compared on social capital satisfaction, self-esteem, and depression.

### 3.2. Measurement Model

The validity of the proposed model was examined using confirmatory factor analysis and structural model analysis. The convergent and discrimination validity of the scales used in the study were also examined [54]. As shown in Table 3, factor loadings (λ) indicating convergent validity ranged from 0.65 to 0.91; these values are above the acceptable threshold (i.e., 0.50). In addition, construct reliability (CR) values ranged from 0.80 to 0.93; these values are also above the acceptable threshold (i.e., 0.70). Similarly, average variance extracted (AVE) values, which ranged between 0.61 and 0.82, are also above the acceptable threshold (i.e., 0.50) [53].

Correlation analysis was used to examine the discriminant validity of the latent variables (Table 3) measured by the scales used in this study. The R square (i.e., R^2^) value between two latent variables must be smaller than the AVE values of the two latent variables for a scale to be considered valid [53]. Table 4 shows that all the determination coefficients were less than the respective AVE values; therefore, the current measurement model can be described as demonstrating sufficient discriminant validity.

### 3.3. Structural Model

**Figure 1:**
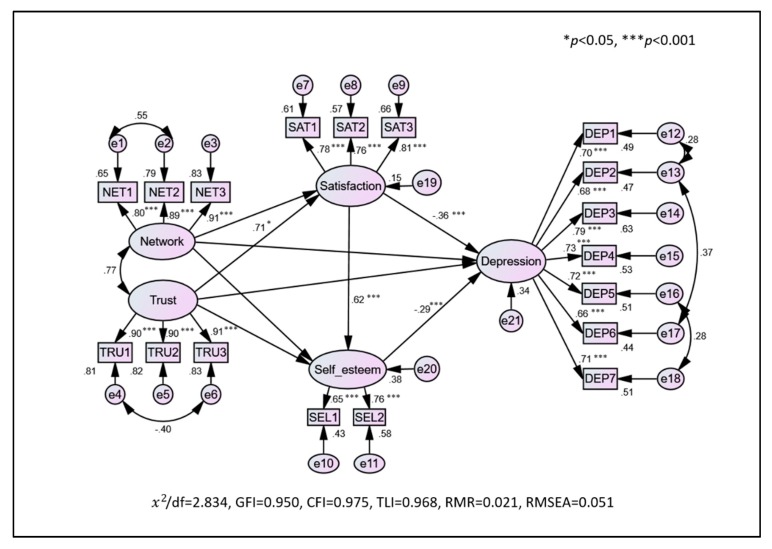
Model displaying factors associated with depression among elderly urban residents in Seoul, Korea (*N* = 701). NET1: There are special people (friends or neighbors) who can help in an emergency, NET2: there are special people (friends or neighbors) who make me comfortable, NET3: there are friends or neighbors around to share joy and sorrow, TRU1: my friends or people around me try to help me, TRU2: I can depend on friends or people around when I’m in trouble, TRU3: I can talk to friends or people around me about my problem, SAT1: satisfaction with social relationships, SAT2: Satisfaction with leisure, SAT3: overall satisfaction, SEL1: positive attitude, SEL2: satisfaction with oneself, DEP1: poor appetite, DEP2: doing well, DEP3: depressed, DEP4: burden, DEP5: loneliness, DEP6: happiness, DEP7: sadness. Note. Values represent standardized factor loadings.

Fit indices for the model presented in Figure 1 were found to be acceptable for all four variables, namely, social capital (i.e., network, trust), social capital satisfaction, self-esteem, and depression: χ^2^/df = 2.83, GFI = 0.95, CFI = 0.98, TLI = 0.97, RMR = 0.02, RMSEA = 0.05. Standardized coefficients for the direct paths from trust to social capital satisfaction (β = 0.71, *p* = 0.04), from social capital satisfaction to self-esteem (β = 0.62, *p* = 0.00), from social capital satisfaction to depression (β = −0.36, *p* = 0.00), and from self-esteem to depression (β = −0.29, *p* = 0.00) were significant. On the other hand, the standardized coefficients for all the direct paths from network to social capital satisfaction (β = −0.35, *p* = 0.36), self-esteem (β = −0.34, *p* = 0.37), and depression (β = −0.04, *p* = 0.90) were not statistically significant.

### 3.4. Mediation Analysis

Table 5 shows the results of the analysis that examined the mediating effect of social capital satisfaction and self-esteem on the relationship between social capital (i.e., network, trust) and depression; this analysis was conducted by adopting the bootstrap estimation procedure in AMOS (bootstrap sample of 500, 95% confidence intervals). Since the indirect effect estimates may not conform to a normal distribution, a bootstrapping method was used to calculate the most accurate confidence intervals for indirect effects [55]. The results of the analysis showed that social capital satisfaction fully mediated the relationship between social capital (i.e., trust) and depression (standardized indirect effect = −0.49, *p* = 0.00). Self-esteem partially mediated the relationship between social capital satisfaction and depression (standardized indirect effect = −0.18, *p* = 0.00).

## 4. Discussion

Multi-generational households were found to report the lowest scores on social capital satisfaction, self-esteem, and the highest scores on depression, among the various household types. This result is consistent with our previous findings that families living in multi-generational households report higher levels of stress and depression than those living in other types of households [18]. A possible explanation for this finding could be that generational differences in political orientations or economic power cause conflicts among family members [56,57]. This result suggests that a comprehensive household welfare service is needed to resolve intra-household conflicts. Intergenerational problems and conflicts may not be solved over short periods. However, many institutions in developed countries have created generational integration programs to strengthen ties between the elderly and younger generations [58]. However, since changes in attitudes tend to be limited to specific situations in which the contact occurred, having various contact experiences is important in resolving conflicts among generations [59]. Kaplan suggested that various generational groups should participate together in school-based educational activities that include senior volunteers [60]. Intergenerational education and programs may solve intergenerational conflicts in the family in an aging society [61]. At the same time, generation-independent education and support, which does not transfer elderly care to the family’s duties, as well as policies to expand social capital for the elderly, are needed to alleviate family conflict caused by aging [62,63]. In addition, integrated recreation not only positively influences children’s attitudes toward the elderly, but also positively affects the subjective well-being of the elderly [64].

The results of our study also support past findings that elderly individuals who have poor health and low income tend to obtain higher scores on tests of depressive symptomatology, than those with good health and income that is representative of the middle-class income class [8,15,18,65]. These findings suggest that physical health and economic factors are related to depression in the elderly.

Our results also support past findings that psychological variables, such as low social capital satisfaction and poor self-esteem, negatively affect depression [66,67,68]. With regards to the psychological variables that were included in our model, we found that one of the strongest predictors of depression is social capital. Although the effect of network on depression was not significant, the effect of trust on depression was indirectly significant. Further, social capital satisfaction and self-esteem significantly mediated the relationship between social capital and depression. These findings suggest that social capital does not directly affect depression; instead, its effect on depression is mediated by social capital satisfaction and self-esteem. Although many studies have examined depression in the elderly from a sociodemographic perspective, this study is unique because it also examined the role that psychological variables play in depression among the elderly. These psychological variables were selected based on the findings of our previous study [5], which revealed that social, not physical, activities are related to depression in the elderly. This study is also unique because it examined the mediating role that these psychological variables play in the relationship between social capital and depression. The mediating effects of social capital satisfaction and self-esteem can promote understanding the effect of social capital on the depressive symptoms of elderly urban residents. Satisfaction and self-esteem are not easily changed with short-term interventions because of their cognitive characteristics. However, the social trust associated with the network positively affects satisfaction and self-esteem and may improve depression. In other words, strengthening social capital can help identify psychological mechanisms that can improve depression. Further, these findings suggest that depression in the elderly can be alleviated by improving their social capital resources. Depression among other members of the community can also be reduced by increasing their social capital resources (Jones, 2014).

Trust is a basic concept that reflects whether people are connected to others [69]; it is variously shaped by four specific factors (family, friends, neighbors, and strangers) [70]. Social trust toward social partners is positively related to subjective happiness [71]. Similar to the findings in our study, social trust in previous studies is correlated with social networks with friends or neighbors, as well as with participation in social activities [72,73]. For example, participants in social education programs, such as vocational, cultural art, and liberal arts programs, reported higher social trust levels than did non-participants [74]. Social trust is also associated with various sociodemographic variables, such as marital status, educational level, residential satisfaction, subjective health status, vocational education, and participation in civic education [30,75]. Therefore, sociodemographic characteristics and social participation in the community may provide information that can help identify elderly people who are vulnerable to mental health challenges. Additionally, providing opportunities for social activities with neighbors will also help promote positive mental health among elderly urban residents who lack social capital.

The study findings underscore the importance of the psychological perspective in the provision of welfare services that are aimed at preventing depression in the elderly. In particular, it is necessary to develop a program that strengthens the trust within social capital of those individuals who are vulnerable to mental health problems, such as anxiety and depression. On the basis of our findings, we recommend periodic and long-term welfare policies for supporting trust-based relationships by engaging in open communication with neighbors. For example, our previous study that conducted a three-hour weekly nature-based activity in urban forest near the participants’ residence for 10 weeks with neighbors for the low-income single elderly group reported that the relationships and trust with neighbors improved [5]. Specially, low-income elderly persons who lack opportunities for social participation and economic capital formed new relationships with neighbors, and their stress and depression reduced after they experienced periodic and long-term programs for leisure activities and health promotion [76]. In order to prevent and reduce the depression of elderly urban residents, it is necessary to support the social environment. Consequently, welfare policies for enhancing trust-based relationships with neighbors will help alleviate depression among elderly urban residents by improving their social capital satisfaction and self-esteem.

This study has several limitations. First, although the KOWEPS consists of a wide dataset, only a limited set of responses that were consistent with the research objectives of this study were included in the analysis. Second, sociodemographic variables were not included in the structural model because this study focused on psychological variables. Therefore, future studies are necessary that can expand this model by controlling for relevant sociodemographic variables. In addition, because this study adopted a cross-sectional design, the causality of the relationships between social capital and depression cannot be determined. We suggest an extended analysis on the basis of our study by adopting longitudinal designs.

## 5. Conclusions

The purpose of this study was to examine the role of sociodemographic characteristics and psychological variables in depression among elderly urban residents. We found that two psychological variables, namely, social capital satisfaction and self-esteem, significantly mediated the relationship between social capital and depression. Therefore, in order to improve efforts to prevent depression among elderly urban residents, it is necessary to identify vulnerable groups of elderly individuals and provide them welfare programs that are aimed at improving their trust in social capital.

## Figures and Tables

**Table 1:** Measurements of the variables in this study.

Latent Variable	Definition	Observed Variables	Categories
Depression	Depressive symptomatology felt in daily life during the past week	Poor appetite	(1) Extremely rarely (2) Rarely (3) Sometimes (4) Most of the time
		Doing well	
		Depressed	
		Burden	
		Lonely	
		Happiness	
		Sadness	
Network	Subjective recognition of the quantitative social network	Who can help in an emergency	
		Who can make one comfortable	
		Who can share in joy and sorrow	
Trust	Confidence in social relationships	Can get help	(1) Strongly disagree (2) Disagree (3) Neither (4) Agree (5) Strongly agree
		Can depend on others when in trouble	
		Can talk about problems	
Satisfaction	The extent to which an individual feels satisfaction	Satisfaction with social relationships	
		Satisfaction with leisure	
		Satisfaction as a whole	
Self-esteem	The extent to which an individual’s respects and approves of himself/herself	Positive attitude toward oneself	(1) Strongly disagree (2) Disagree (3) Agree (4) Strongly agree
		Satisfaction with oneself	

**Table 2:** Sociodemographic differences in social capital satisfaction, self-esteem, and depression (N = 701).

Sociodemographic Group	*N*	%	Satisfaction	Self-Esteem	Depression
			M ± SD	M ± SD	M ± SD
Sex					
Men	295	42.1	9.98 ± 1.87	4.90 ± 1.24	15.82 ± 5.98
Women	406	57.9	9.98 ± 1.92	4.78 ± 1.24	18.59 ± 6.79
χ^2^ (*p*)			0.13 (0.99)	7.61 (0.27)	31.35 (0.051)
Age group					
65–70	206	29.4	9.93 ± 1.83	4.74 ± 1.21	10.76 ± 3.84
71–75	198	28.2	10.22 ± 1.85	4.96 ± 1.29	11.59 ± 4.77
76–80	198	28.2	9.92 ± 1.97	4.78 ± 1.27	11.46 ± 4.28
>80	99	14.1	9.72 ± 1.95	4.83 ± 1.13	12.61 ± 4.39
χ^2^ (*p*)			45.47 (0.13)	11.15 (0.89)	64.68 (0.32)
Educational level					
<Primary school	494	70.5	9.92 ± 1.91	4.78 ± 1.24	11.69 ± 4.32
Middle or high school	181	25.8	10.03 ± 1.91	4.91 ± 1.25	11.08 ± 4.50
>College	26	3.7	10.73 ± 1.40	5.23 ± 1.18	9.58 ± 3.00
χ^2^ (*p*)			20.46 (0.67)	17.00 (0.15)	28.12 (0.92)
Household type					
Solitary	245	35	9.76 ± 1.92	4.71 ± 1.18	12.58 ± 4.62
Elderly spouse	453	64.6	10.12 ± 1.85	4.89 ± 1.27	10.81 ± 4.00
Multi-generation	3	0.4	7.33 ± 3.21	4.33 ± 1.53	17.00 ± 9.85
χ^2^ (*p*)			100.78 (0.00 **)	9.38 (0.67)	176.13 (0.00 **)
Health status					
Good	289	41.2	10.45 ± 1.65	5.16 ± 1.24	10.32 ± 3.71
Bad	412	58.8	9.65 ± 1.99	4.60 ± 1.19	12.25 ± 4.59
χ^2^ (*p*)			42.38 (0.00 **)	41.00 (0.00 **)	51.34 (0.00 **)
Income status					
Low-income class	509	72.6	9.78 ± 1.95	4.72 ± 1.24	11.88 ± 4.59
Middle class	192	27.4	10.50 ± 1.63	5.11 ± 1.21	10.32 ± 3.38
χ^2^ (*p*)			27.15 (0.01 *)	20.68 (0.00 **)	39.59 (0.01 *)

* *p* < 0.05; ** *p* < 0.001.

**Table 3:** Results of the confirmatory factor analysis conducted with scales measuring social capital, social capital satisfaction, self-esteem, and depression.

Latent Variable		Item	Convergent Validity			
			λ	OE.	CR	AVE
Social Capital	Network	NET1	0.80	0.38	0.90	0.75
		NET2	0.89	0.22		
		NET3	0.91	0.17		
	Trust	TRU1	0.90	0.18	0.93	0.82
		TRU2	0.90	0.18		
		TRU3	0.91	0.17		
Satisfaction		SAT1	0.78	0.21	0.90	0.75
		SAT2	0.76	0.27		
		SAT3	0.82	0.16		
Self-esteem		SEL1	0.65	0.24	0.80	0.67
		SEL2	0.76	0.26		
Depression		DEP1	0.70	0.36	0.92	0.61
		DEP2	0.68	0.39		
		DEP3	0.79	0.20		
		DEP4	0.73	0.38		
		DEP5	0.72	0.27		
		DEP6	0.66	0.48		
		DEP7	0.71	0.20		

OE = observational error; CR = construct reliability; AVE = average variance extracted.

**Table 4:** Intercorrelations among the latent variables.

Variable		Social Capital		Satisfaction	Self-Esteem	Depression
		Network	Trust			
Social capital	Network	0.75				
	Trust	0.59	0.82			
Social capital satisfaction		0.17	0.15	0.75		
Self-esteem		0.08	0.07	0.39	0.67	
Depression		0.05	0.05	0.29	0.26	0.61

Values presented in the table indicate R Square (R^2^) values. Diagonal text in boldface represents average variance extracted (AVE) values.

**Table 5:** Standardized coefficients for the relationships between social capital (trust, network), social capital satisfaction, self-esteem, and depression.

Variable	Total (Direct, Indirect)			
	Trust	Network	Satisfaction	Self-Esteem
Social capital satisfaction	0.71 * (0.71 *, 0.00)	−0.31 (−0.31, 0.00)		
Self-esteem	0.80 (0.36, 0.44)	−0.53 (−0.34, −0.19)	0.62 ** (0.62 **, 0.00)	
Depression	−0.44 (0.04, −0.49 *)	0.22 (−0.04, 0.27)	−0.54 ** (−0.36 **, −0.18 **)	−0.29 ** (−0.29 **, 0.00)

* *p* < 0.05; ** *p* < 0.01.

## Appendix A

**Table A1:** Correlation coefficients from Pearson correlation analysis between observed variables in depression.

	DEP2	DEP3	DEP4	DEP5	DEP6	DEP7	DEP8	DEP9	DEP10	DEP11
DEP1	−0.629 **	0.551 **	0.537 **	0.486 **	0.484 **	−0.479 **	0.376 **	0.334 **	0.295 **	0.296 **

** <0.01.

**Table A2:** Correlation coefficients from Pearson correlation analysis between observed variables in self-esteem.

	SEL2	SEL3	SEL4	SEL5	SEL6	SEL7	SEL8	SEL9	SEL10
SEL1	0.496 **	0.341 **	0.382 **	0.310 **	0.374 **	0.312 **	0.219 **	0.388 **	0.168 **

** <0.01.
